# Cutaneous signs in SARS‐CoV‐2 infection: a plea for more rigorous peer review in the time of COVID‐19

**DOI:** 10.1111/bjd.19429

**Published:** 2020-12-01

**Authors:** H. Kittler, P. Tschandl, W. Weninger

**Affiliations:** Department of Dermatology Medical University of Vienna Waehringer Guertel 18‐20 1090 Vienna Austria; Department of Dermatology Medical University of Vienna Waehringer Guertel 18‐20 1090 Vienna Austria; Department of Dermatology Medical University of Vienna Waehringer Guertel 18‐20 1090 Vienna Austria

Dear Editor, Many systemic viral infections are accompanied by skin rashes of variable morphology, some of which are pathognomonic for the respective disease. Limited information is available about cutaneous signs in the course of SARS‐CoV‐2 infection. Anecdotal observations and small case series collected from around the globe have described a plethora of skin signs including (among others) Dengue‐like,^1^ urticarial,^2^, ^3^ crusted and papular,^4^ herpetiform^5^ and varicella‐like rashes^6^ and chilblain‐like lesions (‘COVID toes’).^7^

Rather than being specific to COVID‐19, these different manifestations may represent coincidental skin disease in patients infected with SARS‐CoV‐2. Moreover, some of the included patients represented suspect cases that were not tested or tested negative for the virus, at least by polymerase chain reaction. Both the character and the frequency of skin symptoms in COVID‐19 remain obscure. The first reports from China suggested that skin symptoms are rare; however, it is unclear whether there was specific dermatological expertise involved in the examination of these patients.^8^ Of 1099 patients only four (0·2%) developed a rash. Later, Recalcati reported from Lecco, Italy that 18 of 88 patients (20%) with COVID‐19 developed skin symptoms.^2^ However, these 88 patients were taken from a larger sample of 148 patients and the total population of patients infected with SARS‐CoV‐2 in Lecco remains unknown. Owing to these limitations, the prevalence of 20% reported by Recalcati does not represent a reasonable estimate of the frequency of skin symptoms in all patients infected with SARS‐CoV‐2. Despite these objections, this number has been used as a reference in subsequent publications.^3^

A large proportion of reports on skin signs in COVID‐19 underwent expedited review; some of these reports were even accepted by peer‐reviewed journals on the day of submission. Of 259 articles published on COVID‐19 in the top five dermatology journals between 1 January 2020 and 30 June 2020, 142 reported the date of receipt. For these articles, the median time from submission to acceptance was 6 days [interquartile range (IQR) 14 days] and 12 articles (8·5%) were reported to have been accepted on the day of submission. By comparison, only 4·9% (*n* = 21) of other articles were reported to have been accepted on the day of submission. The median time from submission to acceptance of articles that covered topics other than COVID‐19 (*n* = 431) and that were published during the same time period and in the same journal was significantly longer (median 33 days, IQR 49·5 days; *P* < 0·001) (Figure 1). This has also been observed in other fields of medicine, not just dermatology. As of 30 June 2020, a PubMed search entering the keyword ‘COVID‐19’ yields 25 428 articles published in 2020. Of all publications that provided valid publication history timelines in PubMed (*n* = 5150), no revision was reported in 54·4% (*n* = 2800). The median time from submission to acceptance was 16 days (IQR 24 days) for ‘COVID‐19’ studies, which was significantly shorter than for articles published in the same time period under the keywords ‘H1N1’ (median 34 days, IQR 49 days; *P* < 0·001), ‘melanoma’ (42 days, IQR 44 days; *P* < 0·001) and ‘psoriasis’ (median 33 days, IQR 42·5 days; *P* < 0·001).

**Figure 1:**
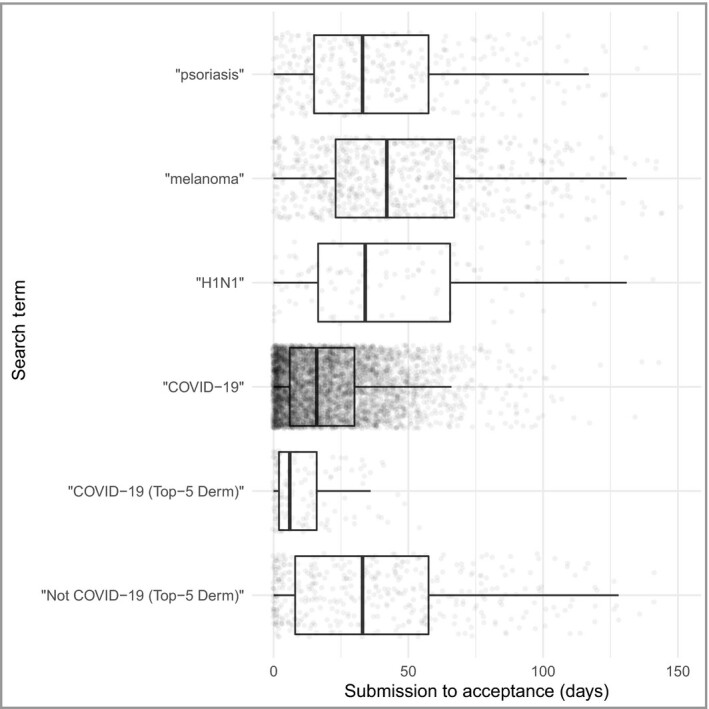
Time from submission to acceptance. Time from submission to acceptance for all PubMed articles containing the keyword denoted on the y‐axis. Top‐5 Derm, the top five dermatology journals according to impact factor (*Journal of the American Academy of Dermatology* or *JAMA Dermatology* or *Journal of Investigative Dermatology* or *British Journal of Dermatology* or *Journal of the European Academy of Dermatology and Venereology*).

We assume that under normal circumstances these studies would not have been published so quickly, if at all. Peer review is lengthy and time‐consuming. Reviewers ask critical questions, but they also provide valuable feedback, which helps researchers to improve their manuscripts before publication. If this control mechanism is suspended, the result may be unfiltered news that may mislead the medical community and unsettle clinicians and patients alike. Indeed, recent reports of ‘COVID toes’ as a sign of viral infection, and the emergence of Kawasaki syndrome‐like disease in children, has resulted in heated debates in the dermatology community. We acknowledge that in a time of an unprecedented health crisis there is a need for a more immediate exchange of information, even if it is of uncertain significance. We believe that preprint servers such as bioRxiv and medRxiv are more suitable for this type of information rather than peer‐reviewed journals.

What we need now is a cooling‐down phase. The time will come when the dust has settled, and we will get a clearer view on the specificity of skin symptoms in patients infected with SARS‐CoV‐2. We appeal to both the researchers who seek the opportunity to publish in high‐impact journals, and the competitive journals and editors who seek the opportunity to attract attention. Leave the hype to the mass media and the propagation of immediate unfiltered news to Twitter and company. Do not suspend peer review!

## Author Contribution

**Harald Kittler:** Writing‐original draft (equal); Writing‐review & editing (equal). **Philipp Tschandl:** Writing‐original draft (equal); Writing‐review & editing (equal). **Wolfgang Weninger:** Writing‐original draft (equal); Writing‐review & editing (equal).
